# Patient Experience of Clinician Compassion Is Associated With Healthcare System Distrust Among Emergency Department Patients

**DOI:** 10.1111/acem.70250

**Published:** 2026-02-25

**Authors:** Patrice Baptista, Cameron Gaines, Christopher W. Jones, Lauren Remboski, Clifford M. Marks, Andrew Nyce, Amanda M. Scudder, Adrian D. Haimovich, Nathan I. Shapiro, Stephen Trzeciak, Brian W. Roberts

**Affiliations:** ^1^ The Department of Emergency Medicine, Cooper University Health Care (CUHC) Cooper Medical School of Rowan University (CMSRU) Camden New Jersey USA; ^2^ The Department of Emergency Medicine Beth Israel Deaconess Medical Center Boston Massachusetts USA; ^3^ The Department of Medicine CUHC/CMSRU Camden New Jersey USA; ^4^ Center for Humanism Cooper Medical School of Rowan University Camden New Jersey USA

## Abstract

**Background:**

Healthcare system distrust—patients' belief that the healthcare system may not act in their best interests—is a recognized social determinant of health and is associated with poor health outcomes, decreased adherence to treatment, and heightened health disparities, especially among marginalized populations. Compassion from clinicians may be a modifiable factor that can foster trust in healthcare systems, but its association with system‐level distrust, particularly in emergency department (ED) settings, remains underexplored.

**Methods:**

We conducted a nested cross‐sectional study enrolling adult patients treated at two urban academic EDs in the United States between September 2023 to May 2024. We separately measured patient experience of physician and nursing staff compassion using the validated 5‐item compassion measure, and patient healthcare system distrust using the Healthcare System Distrust Scale. Multivariable linear regression models, adjusted for demographics and study site, tested associations between perceived compassion and distrust, including subgroup analyses by race, gender, and other sociodemographic factors.

**Results:**

The primary analysis included 779 patients. Both physician (median score 20 [IQR 17–20]) and nursing staff compassion (median score 20 [IQR 17–20]) were highly rated. Higher compassion scores for both physicians (β = −0.62, 95% CI 0.80 to −0.44) and nursing staff (β = −0.24, 95% CI 0.38 to −0.09) were independently associated with lower healthcare system distrust. Compared to non‐Hispanic White patients, Black patients reported higher healthcare system distrust, driven by values (i.e., honesty, motives, and equity)‐based distrust rather than competency‐based distrust, but did not report lower compassion scores. The association between compassion and reduced distrust was consistent across demographic subgroups.

**Conclusion:**

Greater experience of compassion from ED physicians and nursing staff is independently associated with lower healthcare system distrust. Interventions to enhance clinician compassion have the potential to foster trust and may reduce health disparities in emergency care settings.

## 1 Introduction

Healthcare system distrust, the belief that the healthcare system cannot be relied upon to act in the best interests of patients, is a social determinant of health [1]. For example, healthcare system distrust has been identified as a barrier to cancer screening [2], and is associated with increased utilization in unproven treatments [3, 4], and decreased adherence to recommended medical therapy [5, 6]. Distrust has previously been described to consist of two aspects; distrust that the system is not capable of doing what is best (competence distrust) and distrust that the system does not want to do what is best (value distrust) [7]. Further, distrust in the healthcare system may be greater among historically disadvantaged populations (e.g., Black patients) and so could exacerbate existing health disparities [2, 8]. Thus, identifying modifiable factors to increase patient trust is of the utmost importance.

Compassion, defined as the emotional response to another's pain or suffering involving an authentic desire to help [9, 10], has been increasingly recognized as a critical component of patient‐centered care and is associated with improved health outcomes [10]. Specifically, in the emergency department (ED), compassion is associated with decreased patient fear, anxiety, and stigma [11, 12, 13]. We propose that healthcare system distrust is in part due to patients experiencing a lack of compassion from clinicians, particularly among disadvantaged patients. Thus, increasing patients' experience of compassion in the ED may foster trust in the healthcare system.

Our study objectives were, (1) to test for an association between patient experience of compassion from clinicians and healthcare system distrust in the ED, and (2) test for differences in this association between patient populations (e.g., gender and race). We hypothesize that patient experience of compassion would be inversely associated with healthcare system distrust across difference patient populations.

## 2 Methods

### 2.1 Setting

This nested cross‐sectional study was conducted in two urban academic emergency departments in the U.S. [Cooper University Health Care (CUHC), Camden, New Jersey; and Beth Israel Deaconess Medical Center (BIDMC), Boston, Massachusetts] [14]. The study took place from September 2023 to May 2024. The Institutional Review Board at each participating institution approved this study and all subjects provided written informed consent prior to participation. This study is reported in accordance with the Strengthening the Reporting of Observational Studies in Epidemiology (STROBE) Statement (Table S1) [15], and the Checklist for Reporting Of Survey Studies (CROSS) (Table S2) [16].

### 2.2 Study Population and Survey Administration

We enrolled a convenience sample of adult patients who were evaluated in the EDs of the participating institutions. Inclusion criteria were: (1) age 18 years or older; (2) presenting as a patient to the ED; and (3) English or Spanish speaking. Exclusion criteria included: (1) found to have an acute psychiatric emergency (e.g., active suicidal ideation or psychosis), (2) unable to participate in the research questionnaires (i.e., history of dementia, critically ill); and (3) previously participated in the study. Subjects were enrolled at CUHC between the hours of 2 pm to 9 pm, 7 days per week, and at BIDMC between the hours of 8 am to 10 pm on weekdays when research assistants staff the ED. Specific to this study, research assistants received didactic lectures and one‐on‐one training on screening, enrollment, and survey administration from the study investigators [12]. While in the ED, research assistants screened electronic medical record, EPIC (EPIC Systems Corporation, WI) and WebOMR (Beth Israel Lahey Health, MA), for potential eligible subjects at both sites.

### 2.3 Data Collection

Research assistants approached potential subjects for enrollment after completion of care by the ED clinician (i.e., either time of hospital admission or discharge home from the ED for patients not admitted to the hospital). After obtaining written informed consent, subjects were given a computer tablet, which contained the research questionnaire in electronic form. The Research Electronic Data Capture (REDCap) survey distribution tool was used to administer the research questionnaire and capture responses directly into the research database. REDCap is a secure, web‐based software platform designed to support data capture for research studies [17, 18]. Research assistants were instructed to inform the subjects that only the research team will have access to questionnaire answers; specifically, answers will not be shared with the hospital staff involved in their clinical care. After handing the patient the questionnaire, research assistants were instructed to leave the patients' bedside to allow for privacy while filling out the questionnaire, unless the patient requested they stay to help with the questionnaire (e.g., patient required questions to be read to them), and to return in approximately 15 min to collect the computer tablet [14].

The research questionnaire assessed patient experience of ED compassion using the 5‐item compassion measure, a patient‐assessed measure of healthcare compassion. The 5‐item compassion measure has previously been psychometrically validated for use in the ED, out‐patient, and in‐patient settings and consists of five items measured on a four‐point Likert scale [19, 20, 21]. As previously validated, we asked the 5‐item compassion measure in regards to the treating physicians, as well as the nursing staff (Figures S1 and S2) [19]. In cases where patients were treated by more than one physician or nurse, patients were asked to score the overall experience of compassion from all physicians and all nurses who participated in their care. Scores for the five items were summed separately to obtain two composite scores: (1) physician compassion and (2) nursing staff compassion. Potential scores range from 5 to 20 with higher scores indicating greater compassion. The research questionnaire also collected patient responses on gender identity (male, female, transgender male, transgender female, non‐binary, not listed), race (White, Black or African American, Asian, American Indian or Alaska Native, Native Hawaiian or Other Pacific Islander, not listed), ethnicity (Hispanic or Latino), sexual orientation (homosexual, heterosexual, bisexual, asexual, not listed), level of education (did not graduate high school; high school graduate or general educational development [GED]; some college or 2 year degree; 4 year college graduate; more than 4 year college degree), and estimated annual household income.

Using a standardized data collection form, we collected patient age, ED length of stay, and disposition from the emergency department (discharge vs. observation/hospital admission) from the medical record.

### 2.4 Primary Outcome Measure

The primary outcome measure was healthcare system distrust measured using the Healthcare System Distrust Scale (Figure S3), a previously validated, 9‐item assessment of patient distrust of the healthcare system. Items are scored on a 5‐point Likert scale from “Strongly Agree” to “Strongly Disagree.” We summed the scores for each individual item to obtain a composite score (range 9 to 45, higher score indicating greater distrust) [7, 8, 22]. The scale consists of two subscales, (1) four items measuring competence distrust and (2) five items measuring values (i.e., honesty, motives, and equity) distrust [7, 23]. We also summed the scores for each subscale to obtain composite scores (competence range 4 to 20 and values range 5 to 25) [7, 23]. We entered all data into REDCap, and exported into Stata/SE 18.0 for Mac, StataCorp LP (College Station, TX, USA) for analysis.

### 2.5 Data Analysis

We report continuous variables as median and interquartile range (IQR), and categorical variables as frequencies and percentages. We tested the internal reliability of the physician and nursing staff 5‐item compassion measures and the Healthcare System Distrust total scale and subscales using Cronbach's alpha. We display the full distribution of the composite 5‐item compassion measure scores and Healthcare System Distrust scale in histogram form.

We tested for differences in the 5‐item compassion measures by patient demographics using multivariable linear regression models. We entered race (non‐Hispanic White, Black, Asian, other), ethnicity (non‐Hispanic vs. Hispanic), gender (male vs. female vs. transgender/non‐binary/not listed), sexual orientation (heterosexual vs. homosexual/bisexual/asexual/not listed), and level of education as independent variables. We adjusted the model for the fixed effects of site of enrollment. We repeated this analysis for the healthcare system distrust total score, as well as for each subscale.

For the primary analysis we used multivariable linear regression analyses to test if patient experience of physician and nursing staff compassion (i.e., 5‐item compassion measure composite scores) are independently associated with patient Healthcare System Distrust total scale (as well as the subscales). We adjusted the models for the fixed effects of site of enrollment, patient age [24], and ED length of stay. We then performed a sensitivity analysis entering reported income into the primary model (Healthcare System Distrust total score). We also repeated the primary analysis using structural equation modeling to perform a multivariable linear regression model using full information maximum likelihood estimation to allow patients with missing questionnaire data to be included [25].

The five‐item compassion measure has been previously validated to distinctly measure physician and nursing staff compassion; however, we tested for multicollinearity between the scores using variance inflation factor (VIF). Multicollinearity occurs when independent variables are correlated (i.e., may be measuring the same or overlapping constructs) resulting in unstable regression models. A VIF < 5 indicates multicollinearity is not a problem.

We performed subgroup analyses to test if the association between physician and nurse compassion and healthcare distrust varied among different demographics. We used multivariable linear regression to test the association between physician and nursing staff compassion and Healthcare System Distrust scale among the following subgroups: gender (female, male), race and ethnicity (non‐Hispanic White, Black, Asian, Hispanic), sexual orientation (heterosexual, homosexual/bisexual/asexual/not listed), education level (did not graduate high school, high school graduate or GED, some college or college graduate), and ED disposition (discharged vs. observation/admission). We adjusted each model for site of enrollment and age. Given the small number of transgender, non‐binary, and gender not listed individuals, we were unable to perform a subgroup analysis for these groups.

Given the ordinal variables of interest (i.e., 5‐item compassion measures and Healthcare System Distrust scale) have a wide range of possible scores we treated these variables as continuous data and used conservative robust standard errors to estimate the 95% confidence intervals to reduce the risk of type I error for all regression models described.

### 2.6 Sample Size Calculation

For our primary analysis testing physician compassion and nursing staff compassion association with healthcare system distrust, we adjusted the set alpha for multiple comparisons using Bonferroni correction; α = 0.05/2 comparisons = 0.025. Assuming a conservative standard deviation of 2 for the 5‐item compassion measures and 7 for the Health Care Distrust scale, to detect a β coefficient = −0.5 (for every one‐point increase in the 5‐item compassion score the Health Care Distrust scale decreases 0.5 points) with a power = 0.8, we would require 459 subjects. We anticipated missing questionnaire responses for 15% of subjects and planned to enroll a minimum of 528 subjects. For our exploratory models testing the association between patient demographics and healthcare system distrust and patient experience of compassion, as well as subgroup analyses, we used an alpha of 0.05 for statistical significance.

## 3 Results

**FIGURE 1:**
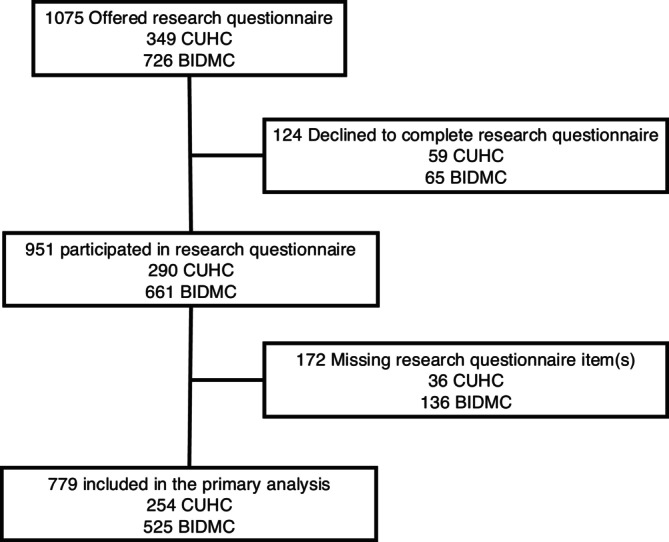
Study flow diagram. BIDMC, Beth Israel Deaconess Medical Center; CUHC, Cooper University Health Care.

A total of 951 patients completed the research questionnaires, and 172 (18.1%) had missing responses, resulting in 779 patients included in the primary analysis (Figure 1). Patient age and self‐reported baseline demographics at the time of presentation to the ED for all patients in the cohort are displayed in (Table 1). Patient demographics were similar between the entire cohort and those included in the primary analysis. The median (IQR) ED length of stay for the entire cohort (*n* = 951) was 9.4 (6.2 to 17.1) hours and for those with complete responses (*n* = 779) was 9.6 (6.3 to 17.5) hours. Among the entire cohort, 442 (46.5%) and among those with complete responses, 352 (45.2%) were discharged from the ED. The remainder of patients were admitted to the hospital.

**TABLE 1:** Patient age and self‐reported baseline patient characteristics.

Variable	All subjects *n* = 951	Included in primary analysis *n* = 779
Age [years (IQR)]	53 (34 to 66)	51 (33 to 65)
Gender [*n* (%)]		
Male	378 (39.8)	321 (41.2)
Female	548 (57.6)	441 (56.6)
Transgender female	1 (0.1)	1 (0.1)
Transgender male	0	0
Non‐binary	7 (0.7)	7 (0.9)
Not listed	2 (0.2)	1 (0.1)
Did not answer	15 (1.6)	8 (1.0)
Race [*n* (%)]		
White/Caucasian	598 (62.9)	498 (63.9)
Black/African American	209 (22.0)	174 (22.3)
Asian	40 (4.2)	30 (3.9)
Other	87 (9.2)	66 (8.5)
Unknown	17 (1.8)	11 (1.4)
Hispanic Ethnicity [*n* (%)]	121 (12.7)	99 (12.7)
Sexual orientation [*n* (%)]		
Heterosexual	732 (77.0)	611 (78.4)
Homosexual	49 (5.2)	44 (5.7)
Bisexual	45 (4.7)	40 (5.1)
Asexual	9 (1.0)	8 (1.0)
Not listed	35 (3.7)	25 (3.2)
Did not answer	81 (8.5)	51 (6.7)
Education [*n* (%)]		
Did not graduate high school	60 (6.3)	49 (6.3)
High school graduate or GED	235 (24.7)	198 (25.4)
Some college or 2 year degree	262 (27.6)	214 (27.5)
4 year college graduate	200 (21.0)	165 (21.2)
More than 4 year college degree	165 (17.4)	135 (17.3)
Did not answer	29 (3.1)	18 (2.3)
Reported income ($1000)	70 (30 to 140)	70 (30 to 140)
[median (IQR)]	*n* = 525	*n* = 446

The median (IQR) physician 5‐item compassion measure score was 20 (17–20) and the nursing staff 5‐item compassion measure score was 20 (17–20). Sixty‐three (6.6%) patients were missing a response to at least one physician 5‐item compassion measure item and 32 (3.4%) patients were missing a response to at least one nursing staff 5‐item compassion measure item. The distributions of the physician and nursing staff 5‐item compassion measure scores are displayed in Figures S4 and S5. Both 5‐item physician and nursing staff compassion measures ranged the full scale from 5 (lowest perceived compassion) to 20 (highest perceived compassion). Both 5‐item compassion measures had excellent internal reliability (physician Cronbach's alpha = 0.91 and nursing staff Cronbach's alpha = 0.95). We found transgender/non‐binary individuals reported less physician compassion compared to males [β = −3.15 (95% CI −6.14 to −0.17)] and females [β = −3.39 (95% CI −6.35 to −0.42)] (Table S3). We did not identify significant differences in the physician or nursing staff 5‐item compassion measures by race/ethnicity or sexual orientation (Tables S3 and S4).

The median [IQR] Health Care Distrust scale was 19 (14 to 24), with higher scores denoting greater distrust. One hundred and sixteen (12.2%) patients were missing a response to at least one Health Care Distrust scale item. The distribution of the Health Care Distrust total scale, as well as the two subscales, is displayed in Figures S6–S8. The Health Care Distrust scale had good internal reliability among the included cohort (Cronbach's alpha, total score = 0.86, competence subscale = 0.78, values subscale = 0.80). Using multivariable regression analysis, we found Black patients reported greater healthcare system distrust [β = 1.35 (95% CI 0.04 to 2.66)] compared to White, non‐Hispanic patients; transgender/non‐binary individuals reported greater healthcare system distrust compared to males [β = 6.10 (95% CI 3.18 to 9.03)] and females [β = 6.08 (95% CI 3.19 to 8.96)]; and high school and college graduates had greater healthcare system distrust compared to those who did not graduate high school (Table S5). We found higher scores for the values‐based distrust subscale among Black patients compared to White, non‐Hispanic patients [β = 0.95 (95% CI 0.14 to 1.75)], but no difference in the competence‐based subscale [β = 0.47 (95% CI 0.10 to 1.05)] (Tables S6 and S7). Similarly, among transgender/non‐binary individuals, we found higher scores for the values‐based distrust subscale, but not the competence‐based subscale compared to males and females (Tables S6 and S7).

We found patient experience of greater physician and nursing staff compassion were both independently associated with lower healthcare system distrust [β = −0.62 (95% CI 0.80 to −0.44) and β = −0.24 (95% CI 0.38 to −0.09), respectively] (Table 2), suggesting every one‐point increase in the physician 5‐item compassion measure score is associated with a 0.62‐point decrease in the Healthcare System Distrust scale and every one‐point increase in the nursing staff 5‐item compassion measure score is associated with a 0.24‐point decrease in the Healthcare System Distrust scale. These results remained consistent after adjusting for reported income (Table S8), as well as when full information maximum likelihood estimation was used to allow patients with missing questionnaire data to be included (Table S9). Model diagnostics did not find evidence of physician and nursing staff compassion multicollinearity (physician compassion VIF = 1.35, nursing staff compassion VIF = 1.34); providing evidence that we successfully measured physician and nursing staff compassion separately and that the two measures were not simply measuring the same construct (e.g., both measuring overall compassion). We found physician compassion to be associated with both the value and competence subscales, and nursing staff compassion to only be associated with the competence subscale (Tables S10 and S11).

**TABLE 2:** Multivariable linear regression results. Healthcare System Distrust scale as the dependent variable.

Variables	β coefficients	95% CI	*p*
Physician compassion^a^	−0.62	−0.80 to −0.44	< 0.001
Nursing staff compassion^a^	−0.24	−0.38 to −0.09	0.001
Age (years)	−0.02	−0.04 to 0.01	0.122
ED length of stay (hours)	−0.00	−0.02 to 0.01	0.601
Site of enrollment			
CUHC	Reference		
BIDMC	0.06	−0.91 to 1.02	0.909

Abbreviations: BIDMC, Beth Israel Deaconess Medical Center; CUHC, Cooper University Health Care; ED, emergency department.

^a^ Every one‐point increase in the 5‐item compassion measure composite score.

**FIGURE 2:**
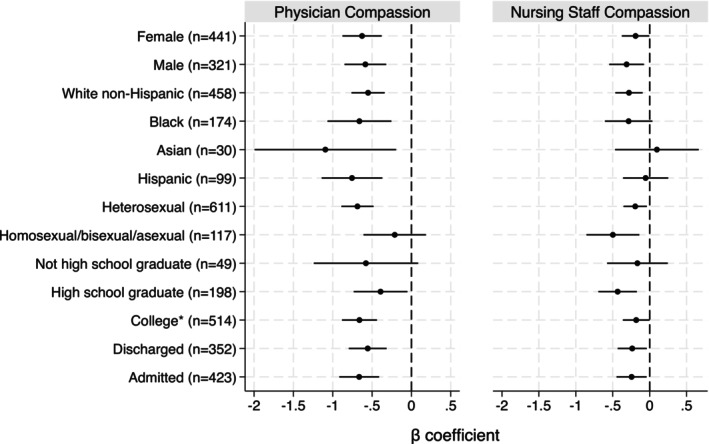
Results of subgroup analyses. Multivariable linear regression models with Healthcare System Distrust total scale as the dependent variable and the physician 5‐item compassion measure and nursing staff 5‐item compassion measure as the independent variables of interest. All models are adjusted for site of enrollment and patient age. To the left of the vertical dashed line indicates higher 5‐item compassion measure is associated with lower Healthcare System Distrust scale. Horizontal lines indicate 95% confidence intervals for the β coefficient. *Attended some college, 4 year college graduate, or more than 4 year college degree.

We found physician and nursing staff compassion were both independently associated with healthcare system distrust across multiple subgroups (Figure 2). Physician compassion had a stronger association with healthcare system distrust than nursing staff compassion among most subgroups.

## 4 Discussion

In this cross‐sectional study, we found patient experience of greater physician and nursing staff compassion in the ED was independently associated with lower healthcare system distrust. These findings suggest a critical role of patients' experience of compassion in fostering trust in the healthcare system, specifically in ED settings where patient interactions are often limited, high‐stakes, and accompanied by patient stress [26]. These results are consistent with prior research demonstrating an association between patient experience of compassion in the ED and fewer post‐traumatic stress disorder (PTSD) symptoms 30 days after discharge among patients who experience a medical emergency [11]. Similarly, among patients with opioid use disorder, greater compassion from ED staff is associated with less fear of stigma [12]. It is possible that compassion in the ED reduces patients' fear and anxiety during these stressful encounters, thus improving patients' perception of the healthcare system as a whole. These findings provide rationale for future research to identify clinician actions that increase the patient experience of compassion, as well as barriers to compassion. For example, prior research has found clinicians identify barriers to compassion within the healthcare system (e.g., organizational demands, workplace stresses, and lack of support) [27].

We also found Black individuals and transgender/binary individuals, historically marginalized populations, reported greater healthcare system distrust. Consistent with prior research, these differences were driven by differences in values distrust as opposed to competence distrust [8, 23]. Physician compassion appears to have a stronger correlation with healthcare system distrust compared to nursing staff compassion. This association remained consistent across multiple subgroup analyses. While physician compassion was associated with lower values and competence distrust ratings, nursing compassion was only associated with less competence distrust. Prior research has suggested values distrust among Black individuals is driven by concerns about experimentation and lying to make money [23]. Physicians are often viewed as the key representatives of the healthcare system, so the perception of greater physician compassion may lead patients to view the broader health care system more favorably [28]. This may also explain why physician compassion had a stronger association with healthcare system distrust than nursing staff compassion across most subgroups. We also found that both physician and nursing compassion were associated with lower competence distrust, consistent with prior research showing an association between patient perception of greater clinician empathy and trust in clinician competence [29].

Our results differ from previous studies which have reported disparities in patient experiences of communication and being treated with disrespect based on race [30, 31]. Although Black patients in our cohort reported greater healthcare system distrust, we did not identify significant racial or ethnic differences in experience of compassion from clinicians. Racial disparities in distrust have previously been shown to be driven by prior experiences of racial discrimination [8]. It is unlikely that compassion at the point of care could account for differences in distrust rooted in prior experiences of discrimination. However, we did find the association between physician compassion and healthcare system distrust to be consistent across racial and ethnic subgroups, warranting future testing of compassion at the point of care as a potential intervention to reduce healthcare system distrust.

There are several limitations of our study which should be acknowledged. First, our study relied on a convenience sample of patients presenting to the enrollment EDs, which may limit the generalizability of our findings to other geographically located EDs. Similarly, only English and Spanish speaking subjects were included. Second, missing data for both the 5‐item compassion measure and Healthcare System Distrust scale could introduce bias, although sensitivity analyses confirmed the robustness of our results. Third, our reliance on patient‐reported measures introduces the possibility of recall bias or response bias. We attempted to mitigate this risk by administering the research questionnaire while patients were still in the ED. Further, both scales utilized have been previously validated, and our results found the internal reliability for both measures to be consistent with prior studies [7, 12, 19, 21, 23]. Fourth, given the study design we cannot infer causation. While we hypothesized that greater experience of compassion at the point of care attenuates healthcare system distrust, an alternative explanation for our findings is that patients' baseline levels of healthcare system distrust may influence how they experience compassion. Similarly, while this study evaluated the association between compassion and healthcare system distrust during a single patient encounter in the ED, future research is needed to test if this association is present in other healthcare settings and if repeated exposure to clinician compassion reduces healthcare system distrust over time. For example, longitudinal studies in different health care settings such as primary care could provide valuable insights into the directionality and causality of this relationship, offering a deeper understanding of how experiences of compassion and healthcare system distrust interact over time. Fifth, we found transgender/non‐binary individuals reported experiencing less compassion and greater healthcare distrust; however, the small number of these patients prevented subgroup analysis of the association between compassion and distrust in this cohort. Therefore, we suggest this is an important population for future research. Sixth, we did not evaluate patient trust in their individual clinicians, nor did we assess clinician characteristics (e.g., clinician age, gender, race/ethnicity) and how such characteristics affect patient experience of compassion and healthcare system distrust. Future research is needed to test the impact of clinician characteristics and if patient trust in their treating physician mediates the association between compassion and healthcare system distrust. Finally, the high compassion scores noted at both sites create a potential ceiling effect, which can limit our ability to detect differences in compassion.

## 5 Conclusion

In conclusion, patient experience of clinician compassion in the ED is associated with healthcare system distrust. Future research is warranted to test if interventions aimed at cultivating compassion among ED physicians and nursing staff enhance patient trust in the healthcare system and improve healthcare equity.

## Author Contributions

All authors have made substantial contributions to this paper: B.W.R. supervised all aspects of the study and takes responsibility for the paper as a whole. B.W.R., S.T., P.B., and A.N. conceived this study. C.W.J., L.B., C.G., C.M.M., A.M.S., and A.D.H. acquired the data. B.W.R., P.B., C.G., L.B., C.M.M., A.M.S., and A.D.H. managed the data. B.W.R., C.M.M., and N.I.S. analyzed the data and interpreted results. B.W.R., C.M.M., S.T., and N.I.S. drafted the manuscript, and all authors contributed substantially to its revision. All authors approved the manuscript in its final form.

## Funding

This work was supported by T. Denny Sanford Institute for Empathy and Compassion at University of California San Diego.

## Conflicts of Interest

Dr. Trzeciak is co‐author of two books on compassion. He donates the book proceeds to the Cooper Foundation. He has received payments for speaking engagements related to the books. Dr. Jones has no competing interests related to this work, though he has been an investigator on studies funded by AstraZeneca, Vapotherm, Abbott, and Ophirex. None of the other authors have conflicts of interest to disclose.

## Supporting information


**Data S1:** Supporting Information.

## Data Availability

The data that support the findings of this study will be openly available at Roberts, Brian (2025), “DBAD”, Mendeley Data, V1, doi: https://doi.org/10.17632/tp5vd9k53s.1.
