# Supplementary material for: Patient Experience of Clinician Compassion Is Associated With Healthcare System Distrust Among Emergency Department Patients
Source: Acad Emerg Med. 2026 Feb 25;33(2):e70250. doi: 10.1111/acem.70250 (PMC12933280; doi:10.1111/acem.70250)
Supplement: Supplementary file 1 — Data S1: Supporting Information. [file ACEM-33-0-s001.docx]

**Patient experience of clinician compassion is associated with healthcare system distrust among emergency department patients**

**Supplemental Material**

Patrice Baptista, MD^1^; Cameron Gaines, BA^1^; Christopher W. Jones, MD^1^;

Lauren Remboski, MS^1^; Clifford M. Marks, MD, MBA^2^; Andrew Nyce, MD^1^;

Amanda M. Scudder, MD^2^; Adrian D. Haimovich, MD, PhD^2^; Nathan I. Shapiro, MD, MPH^2^;

Stephen Trzeciak, MD, MPH^3,4^; Brian W. Roberts, MD, MSc^1,4^

1: The Department of Emergency Medicine, Cooper University Health Care (CUHC), Cooper Medical School of Rowan University (CMSRU), Camden, New Jersey, USA

2: The Department of Emergency Medicine, Beth Israel Deaconess Medical Center, Boston, Massachusetts, USA

3: The Department of Medicine, CUHC/CMSRU, Camden, New Jersey, USA

4: Center for Humanism, Cooper Medical School of Rowan University, Camden, NJ, USA

For Submission to *Academic Emergency Medicine*

Address for correspondence:

Brian W. Roberts, MD, MSc

Department of Emergency Medicine

Cooper University Health Care

Education & Research Building, Second Floor

401 Haddon Avenue

Camden, NJ 08103

roberts-brian-w@cooperhealth.edu

Table of Contents

[I. Research Checklists 3](#_Toc205353974)

[**Supplemental Table 1**: Strengthening the Reporting of Observational Studies in Epidemiology (STROBE) Statement checklist 3](#_Toc205353975)

[**Supplement Table 2:** Checklist for Reporting Of Survey Studies (CROSS) 5](#_Toc205353976)

[II. Supplemental Tables 8](#_Toc205353977)

[**Supplemental Table 3**: Multivariable linear regression model with Physician 5-item compassion measure as the dependent variable 8](#_Toc205353978)

[**Supplemental Table 4**: Multivariable linear regression model with Nursing staff 5-item compassion measure as the dependent variable 9](#_Toc205353979)

[**Supplemental Table 5**: Multivariable linear regression model with Healthcare System Distrust total scale as the dependent variable 10](#_Toc205353980)

[**Supplemental Table 6**: Multivariable linear regression model with Healthcare System Distrust values subscale as the dependent variable 11](#_Toc205353981)

[**Supplemental Table 7**: Multivariable linear regression model with Healthcare System Distrust competence subscale as the dependent variable 12](#_Toc205353982)

[**Supplemental Table 8**: Multivariable linear regression model. Sensitivity analysis with Healthcare System Distrust total scale as the dependent variable entering reported income 13](#_Toc205353983)

[**Supplemental Table 9**: Multivariable linear regression model with Healthcare System Distrust total scale as the dependent variable, using full information maximum likelihood estimation 14](#_Toc205353984)

[**Supplemental Table 10**: Multivariable linear regression model with Healthcare System Distrust values subscale as the dependent variable 15](#_Toc205353985)

[**Supplemental Table 11**: Multivariable linear regression model with Healthcare System Distrust competence subscale as the dependent variable 16](#_Toc205353986)

[III. Supplemental Figures 17](#_Toc205353987)

[**Supplemental Figure 1**: Physician 5-item compassion measure items 17](#_Toc205353988)

[**Supplemental Figure 2**: Nursing staff 5-item compassion measure items. 18](#_Toc205353989)

[**Supplemental Figure 3**: Healthcare System Distrust Scale items 19](#_Toc205353990)

[**Supplemental Figure 4**: Distribution of the physician 5-item compassion measure 20](#_Toc205353991)

[**Supplemental Figure 5**: Distribution of the nursing staff 5-item compassion measure 21](#_Toc205353992)

[**Supplemental Figure 6**: Distribution of the Healthcare System Distrust total scale 22](#_Toc205353993)

[**Supplemental Figure 7**: Distribution of the Healthcare System Distrust values subscale 23](#_Toc205353994)

[**Supplemental Figure 8**: Distribution of the Healthcare System Distrust competence subscale 24](#_Toc205353995)

# I. Research Checklists

**Supplemental Table 1**: Strengthening the Reporting of Observational Studies in Epidemiology (STROBE) Statement checklist.

|  | Item No | Recommendation |
| --- | --- | --- |
| **Title and abstract** | 1 | (*a*) Indicate the study’s design with a commonly used term in the title or the abstract. Title page and page1 |
|  |  | (*b*) Provide in the abstract an informative and balanced summary of what was done and what was found. Page 1 |
| Introduction | | |
| Background/rationale | 2 | Explain the scientific background and rationale for the investigation being reported. Page 3 |
| Objectives | 3 | State specific objectives, including any prespecified hypotheses. Page 3 |
| Methods | | |
| Study design | 4 | Present key elements of study design early in the paper. Page 4 |
| Setting | 5 | Describe the setting, locations, and relevant dates, including periods of recruitment, exposure, follow-up, and data collection. Page 4 |
| Participants | 6 | (*a*) Give the eligibility criteria, and the sources and methods of selection of participants. Describe methods of follow-up. Page 4 |
|  |  | (*b*) For matched studies, give matching criteria and number of exposed and unexposed. N/A |
| Variables | 7 | Clearly define all outcomes, exposures, predictors, potential confounders, and effect modifiers. Give diagnostic criteria, if applicable. Pages 5 and 6 |
| Data sources/ measurement | 8* | For each variable of interest, give sources of data and details of methods of assessment (measurement). Describe comparability of assessment methods if there is more than one group. Pages 5 and 6 |
| Bias | 9 | Describe any efforts to address potential sources of bias. Pages 7 and 8 |
| Study size | 10 | Explain how the study size was arrived at. Page 8 |
| Quantitative variables | 11 | Explain how quantitative variables were handled in the analyses. If applicable, describe which groupings were chosen and why. Pages 7 and 8 |
| Statistical methods | 12 | (*a*) Describe all statistical methods, including those used to control for confounding. Pages 7 and 8 |
|  |  | (*b*) Describe any methods used to examine subgroups and interactions. Page 8 |
|  |  | (*c*) Explain how missing data were addressed. Page 7 |
|  |  | (*d*) If applicable, explain how loss to follow-up was addressed. N/A |
|  |  | (*e*) Describe any sensitivity analyses. Pages 7 and 8 |
| Results | | |
| Participants | 13* | (a) Report numbers of individuals at each stage of study—eg numbers potentially eligible, examined for eligibility, confirmed eligible, included in the study, completing follow-up, and analysed. Figure 1 |
|  |  | (b) Give reasons for non-participation at each stage. Figure 1. |
|  |  | (c) Consider use of a flow diagram. Figure 1. |
| Descriptive data | 14* | (a) Give characteristics of study participants (eg demographic, clinical, social) and information on exposures and potential confounders. Table 1 |
|  |  | (b) Indicate number of participants with missing data for each variable of interest. Table 1 |
|  |  | (c) Summarise follow-up time (eg, average and total amount). N/A |
| Outcome data | 15* | Report numbers of outcome events or summary measures over time. Supplemental material |
| Main results | 16 | (*a*) Give unadjusted estimates and, if applicable, confounder-adjusted estimates and their precision (eg, 95% confidence interval). Make clear which confounders were adjusted for and why they were included. Table 2 |
|  |  | (*b*) Report category boundaries when continuous variables were categorized. N/A |
|  |  | (*c*) If relevant, consider translating estimates of relative risk into absolute risk for a meaningful time period. N/A |
| Other analyses | 17 | Report other analyses done—eg analyses of subgroups and interactions, and sensitivity analyses. Figure 2 |
| Discussion | | |
| Key results | 18 | Summarise key results with reference to study objectives. Page 12 |
| Limitations | 19 | Discuss limitations of the study, taking into account sources of potential bias or imprecision. Discuss both direction and magnitude of any potential bias. Pages 13 and 14 |
| Interpretation | 20 | Give a cautious overall interpretation of results considering objectives, limitations, multiplicity of analyses, results from similar studies, and other relevant evidence. Pages 12-14 |
| Generalisability | 21 | Discuss the generalisability (external validity) of the study results. Pages 12-14 |
| Other information | | |
| Funding | 22 | Give the source of funding and the role of the funders for the present study and, if applicable, for the original study on which the present article is based. Title Page. |

## **Supplement Table 2:** Checklist for Reporting Of Survey Studies (CROSS)

| **Section/topic** | **Item** | **Item description** | **Reported on page #** |
| --- | --- | --- | --- |
| **Title and abstract** | | |  |
| Title and abstract | 1a | State the word “survey” along with a commonly used term in title or abstract to introduce the study’s design. | 1 |
|  | 1b | Provide an informative summary in the abstract, covering background, objectives, methods, findings/results, interpretation/discussion, and conclusions. | 1 |
| **Introduction** | | |  |
| Background | 2 | Provide a background about the rationale of study, what has been previously done, and why this survey is needed. | 3 |
| Purpose/aim | 3 | Identify specific purposes, aims, goals, or objectives of the study. | 3 |
| **Methods** | | |  |
| Study design | 4 | Specify the study design in the methods section with a commonly used term (e.g., cross-sectional or longitudinal). | 4 |
|  | 5a | Describe the questionnaire (e.g., number of sections, number of questions, number and names of instruments used). | 5-6 |
| Data collection methods | 5b | Describe all questionnaire instruments that were used in the survey to measure particular concepts. Report target population, reported validity and reliability information, scoring/classification procedure, and reference links (if any). | 5-6 |
|  | 5c | Provide information on pretesting of the questionnaire, if performed (in the article or in an online supplement). Report the method of pretesting, number of times questionnaire was pre-tested, number and demographics of participants used for pretesting, and the level of similarity of demographics between pre-testing participants and sample population. | 5-6 |
|  | 5d | Questionnaire if possible, should be fully provided (in the article, or as appendices or as an online supplement). | Supplemental material |
| Sample characteristics | 6a | Describe the study population (i.e., background, locations, eligibility criteria for participant inclusion in survey, exclusion criteria). | 4 |
|  | 6b | Describe the sampling techniques used (e.g., single stage or multistage sampling, simple random sampling, stratified sampling, cluster sampling, convenience sampling). Specify the locations of sample participants whenever clustered sampling was applied. | 4 |
|  | 6c | Provide information on sample size, along with details of sample size calculation. | 8 |
|  | 6d | Describe how representative the sample is of the study population (or target population if possible), particularly for population-based surveys. | 4 |
| Survey  administration | 7a | Provide information on modes of questionnaire administration, including the type and number of contacts, the location where the survey was conducted (e.g., outpatient room or by use of online tools, such as SurveyMonkey). | 4 |
|  | 7b | Provide information of survey’s time frame, such as periods of recruitment, exposure, and follow-up days. | N/A |
|  | 7c | Provide information on the entry process:  –>For non-web-based surveys, provide approaches to minimize human error in data entry.  –>For web-based surveys, provide approaches to prevent “multiple participation” of participants. | N/A |
| Study preparation | 8 | Describe any preparation process before conducting the survey (e.g., interviewers’ training process, advertising the survey). | 5-6 |
| Ethical considerations | 9a | Provide information on ethical approval for the survey if obtained, including informed consent, institutional review board [IRB] approval, Helsinki declaration, and good clinical practice [GCP] declaration (as appropriate). | 4 |
|  | 9b | Provide information about survey anonymity and confidentiality and describe what mechanisms were used to protect unauthorized access. | 5-6 |
| Statistical  analysis | 10a | Describe statistical methods and analytical approach. Report the statistical software that was used for data analysis. | 6-7 |
|  | 10b | Report any modification of variables used in the analysis, along with reference (if available). | N/A |
|  | 10c | Report details about how missing data was handled. Include rate of missing items, missing data mechanism (i.e., missing completely at random [MCAR], missing at random [MAR] or missing not at random [MNAR]) and methods used to deal with missing data (e.g., multiple imputation). | 7 |
|  | 10d | State how non-response error was addressed. | 7 |
|  | 10e | For longitudinal surveys, state how loss to follow-up was addressed. | N/A |
|  | 10f | Indicate whether any methods such as weighting of items or propensity scores have been used to adjust for non-representativeness of the sample. | N/A |
|  | 10g | Describe any sensitivity analysis conducted. | 7 |
| **Results** | | |  |
| Respondent characteristics | 11a | Report numbers of individuals at each stage of the study. Consider using a flow diagram, if possible. | Figure 1 |
|  | 11b | Provide reasons for non-participation at each stage, if possible. | Figure 1 |
|  | 11c | Report response rate, present the definition of response rate or the formula used to calculate response rate. | Figure 1 |
|  | 11d | Provide information to define how unique visitors are determined. Report number of unique visitors along with relevant proportions (e.g., view proportion, participation proportion, completion proportion). |  |
| Descriptive  results | 12 | Provide characteristics of study participants, as well as information on potential confounders and assessed outcomes. | Table 1 |
| Main findings | 13a | Give unadjusted estimates and, if applicable, confounder-adjusted estimates along with 95% confidence intervals and p-values. | Table 2 |
|  | 13b | For multivariable analysis, provide information on the model building process, model fit statistics, and model assumptions (as appropriate). | 10 |
|  | 13c | Provide details about any sensitivity analysis performed. If there are considerable amount of missing data, report sensitivity analyses comparing the results of complete cases with that of the imputed dataset (if possible). | 10 |
| **Discussion** | | |  |
| Limitations | 14 | Discuss the limitations of the study, considering sources of potential biases and imprecisions, such as non-representativeness of sample, study design, important uncontrolled confounders. | 13-14 |
| Interpretations | 15 | Give a cautious overall interpretation of results, based on potential biases and imprecisions and suggest areas for future research. | 12 |
| Generalizability | 16 | Discuss the external validity of the results. | 12-14 |
| **Other sections** | | |  |
| Role of funding source | 17 | State whether any funding organization has had any roles in the survey’s design, implementation, and analysis. | Title page |
| Conflict of interest | 18 | Declare any potential conflict of interest. | Title page |
| Acknowledgements | 19 | Provide names of organizations/persons that are acknowledged along with their contribution to the research. | Title page |

# II. Supplemental Tables

**Supplemental Table 3**: Multivariable linear regression model with Physician 5-item compassion measure as the dependent variable (n = 801).

| Variables | β Coefficients | 95% CI | p-value |
| --- | --- | --- | --- |
|  |  |  |  |
| Race |  |  |  |
| White | Reference |  |  |
| Black | -0.44 | -1.03 to 0.15 | 0.140 |
| Asian | -1.13 | -2.48 to 0.21 | 0.099 |
| Other | -0.03 | -0.90 to 0.83 | 0.938 |
| Not Hispanic | Reference |  |  |
| Hispanic | -0.34 | -1.08 to 0.40 | 0.368 |
| Gender |  |  |  |
| Male | Reference |  |  |
| Female | 0.23 | -0.19 to 0.66 | 0.285 |
| Transgender/non-binary/not listed | -3.15 | -6.14 to -0.17 | 0.038 |
| Heterosexual | Reference |  |  |
| Homosexual/bisexual/asexual/not listed | -0.38 | -0.99 to 0.24 | 0.230 |
| Education level |  |  |  |
| Did not graduate high school | Reference |  |  |
| High school graduate or GED | -0.43 | -1.24 to 0.39 | 0.303 |
| Some college or 2-year degree | -0.77 | -1.57 to 0.03 | 0.060 |
| 4-year college graduate | -0.96 | -1.80 to -0.13 | 0.024 |
| More than 4-year college degree | -0.28 | -1.11 to 0.56 | 0.517 |
| Site of enrollment |  |  |  |
| CUHC | Reference |  |  |
| BIDMC | 0.62 | 0.14 to 1.09 | 0.011 |

BIDMC, Beth Israel Deaconess Medical Center; CUHC, Cooper University Health Care

**Supplemental Table 4**: Multivariable linear regression model with Nursing staff 5-item compassion measure as the dependent variable (n = 818).

| Variables | β Coefficients | 95% CI | p-value |
| --- | --- | --- | --- |
|  |  |  |  |
| Race |  |  |  |
| White | Reference |  |  |
| Black | -0.64 | -1.32 to 0.03 | 0.063 |
| Asian | -0.76 | -2.28 to 0.76 | 0.326 |
| Other | 0.46 | -0.38 to 1.30 | 0.284 |
| Not Hispanic | Reference |  |  |
| Hispanic | -0.06 | -0.86 to 0.74 | 0.892 |
| Gender |  |  |  |
| Male | Reference |  |  |
| Female | -0.11 | -0.60 to 0.37 | 0.641 |
| Transgender/non-binary/not listed | -3.54 | -7.55 to 0.47 | 0.084 |
| Heterosexual | Reference |  |  |
| Homosexual/bisexual/asexual/not listed | -0.37 | -1.01 to 0.27 | 0.258 |
| Education level |  |  |  |
| Did not graduate high school | Reference |  |  |
| High school graduate or GED | -0.77 | -1.64 to 0.11 | 0.085 |
| Some college or 2-year degree | -0.79 | -1.63 to 0.04 | 0.064 |
| 4-year college graduate | -0.97 | -1.86 to -0.08 | 0.032 |
| More than 4-year college degree | -0.60 | -1.50 to 0.30 | 0.189 |
| Site of enrollment |  |  |  |
| CUHC | Reference |  |  |
| BIDMC | 0.70 | 0.16 to 1.25 | 0.011 |

BIDMC, Beth Israel Deaconess Medical Center; CUHC, Cooper University Health Care

**Supplemental Table 5**: Multivariable linear regression model with Healthcare System Distrust total scale as the dependent variable (n = 761).

| Variables | β Coefficients | 95% CI | p-value |
| --- | --- | --- | --- |
|  |  |  |  |
| Race |  |  |  |
| White | Reference |  |  |
| Black | 1.35 | 0.04 to 2.66 | 0.044 |
| Asian | 2.10 | -0.42 to 4.61 | 0.102 |
| Other | -0.06 | -1.94 to 1.82 | 0.947 |
| Not Hispanic | Reference |  |  |
| Hispanic | -0.14 | -1.76 to 1.47 | 0.861 |
| Gender |  |  |  |
| Male | Reference |  |  |
| Female | 0.03 | -0.96 to 1.02 | 0.957 |
| Transgender/non-binary/not listed | 6.10 | 3.18 to 9.03 | <0.001 |
| Heterosexual | Reference |  |  |
| Homosexual/bisexual/asexual/not listed | 0.61 | -0.74 to 1.95 | 0.375 |
| Education level |  |  |  |
| Did not graduate high school | Reference |  |  |
| High school graduate or GED | 1.98 | -0.01 to 3.97 | 0.051 |
| Some college or 2-year degree | 3.28 | 1.26 to 5.29 | 0.001 |
| 4-year college graduate | 2.59 | 0.54 to 4.65 | 0.013 |
| More than 4-year college degree | 4.10 | 1.87 to 6.33 | <0.001 |
| Site of enrollment |  |  |  |
| CUHC | Reference |  |  |
| BIDMC | -0.66 | -1.74 to 0.41 | 0.227 |

BIDMC, Beth Israel Deaconess Medical Center; CUHC, Cooper University Health Care

**Supplemental Table 6**: Multivariable linear regression model with Healthcare System Distrust values subscale as the dependent variable (n = 775).

| Variables | β Coefficients | 95% CI | p-value |
| --- | --- | --- | --- |
|  |  |  |  |
| Race |  |  |  |
| White | Reference |  |  |
| Black | 0.95 | 0.14 to 1.75 | 0.022 |
| Asian | 1.47 | -0.12 to 3.05 | 0.070 |
| Other | 0.46 | -0.81 to 1.74 | 0.477 |
| Not Hispanic | Reference |  |  |
| Hispanic | -0.26 | -1.27 to 0.75 | 0.611 |
| Gender |  |  |  |
| Male | Reference |  |  |
| Female | -0.07 | -0.69 to 0.55 | 0.835 |
| Transgender/non-binary/not listed | 4.43 | 2.09 to 6.78 | <0.001 |
| Heterosexual | Reference |  |  |
| Homosexual/bisexual/asexual/not listed | 0.24 | -0.62 to 1.10 | 0.579 |
| Education level |  |  |  |
| Did not graduate high school | Reference |  |  |
| High school graduate or GED | 0.90 | -0.42 to 2.21 | 0.180 |
| Some college or 2-year degree | 1.50 | 0.19 to 2.81 | 0.025 |
| 4-year college graduate | 1.21 | -0.14 to 2.55 | 0.079 |
| More than 4-year college degree | 1.98 | 0.51 to 3.44 | 0.008 |
| Site of enrollment |  |  |  |
| CUHC | Reference |  |  |
| BIDMC | -0.54 | -1.21 to 0.13 | 0.116 |

BIDMC, Beth Israel Deaconess Medical Center; CUHC, Cooper University Health Care

**Supplemental Table 7**: Multivariable linear regression model with Healthcare System Distrust competence subscale as the dependent variable (n = 808).

| Variables | β Coefficients | 95% CI | p-value |
| --- | --- | --- | --- |
|  |  |  |  |
| Race |  |  |  |
| White | Reference |  |  |
| Black | 0.47 | -0.10 to 1.05 | 0.108 |
| Asian | 0.50 | -0.63 to 1.63 | 0.382 |
| Other | -0.61 | -1.40 to 0.17 | 0.125 |
| Not Hispanic | Reference |  |  |
| Hispanic | -0.03 | -0.76 to 0.71 | 0.944 |
| Gender |  |  |  |
| Male | Reference |  |  |
| Female | 0.18 | -0.26 to 0.61 | 0.425 |
| Transgender/non-binary/not listed | 1.77 | -0.03 to 3.57 | 0.054 |
| Heterosexual | Reference |  |  |
| Homosexual/bisexual/asexual/not listed | 0.32 | -0.26 to 0.91 | 0.282 |
| Education level |  |  |  |
| Did not graduate high school | Reference |  |  |
| High school graduate or GED | 1.04 | 0.25 to 1.82 | 0.010 |
| Some college or 2-year degree | 1.70 | 0.91 to 2.50 | <0.001 |
| 4-year college graduate | 1.55 | 0.74 to 2.37 | <0.001 |
| More than 4-year college degree | 2.10 | 1.21 to 2.99 | <0.001 |
| Site of enrollment |  |  |  |
| CUHC | Reference |  |  |
| BIDMC | -0.22 | -0.69 to 0.25 | 0.364 |

BIDMC, Beth Israel Deaconess Medical Center; CUHC, Cooper University Health Care

## **Supplemental Table 8**: Multivariable linear regression model. Sensitivity analysis with Healthcare System Distrust total scale as the dependent variable entering reported income

(n = 445).

| **Variables** | **β Coefficients** | **95% CI** | **p-value** |
| --- | --- | --- | --- |
|  |  |  |  |
| Physician Compassion* | -0.56 | -0.79 to -0.34 | <0.001 |
| Nursing staff compassion* | -0.27 | -0.45 to -0.08 | 0.006 |
| Age (years) | -0.02 | -0.05 to 0.02 | 0.360 |
| ED length of stay (hours) | -0.01 | -0.02 to 0.001 | 0.085 |
| Site of enrollment |  |  |  |
| CUHC | Reference |  |  |
| BIDMC | -0.37 | -1.71 to 0.98 | 0.593 |
| Reported income^†^ | 0.02 | -0.03 to 0.06 | 0.464 |

* Every one-point increase in the 5-item compassion measure composite score.

^†^ Calibrated for increase in United States $10,000.

BIDMC, Beth Israel Deaconess Medical Center; CUHC, Cooper University Health Care; ED, emergency department

**Supplemental Table 9**: Multivariable linear regression model with Healthcare System Distrust total scale as the dependent variable, using full information maximum likelihood estimation to allow patients with missing questionnaire data to be included (n = 951).

| **Variables** | **β Coefficients** | **95% CI** | **p-value** |
| --- | --- | --- | --- |
|  |  |  |  |
| Physician Compassion* | -0.58 | -0.77 to -0.39 | <0.001 |
| Nursing staff compassion* | -0.25 | -0.40 to -0.11 | <0.001 |
| Age (years) | -0.02 | -0.04 to 0.01 | 0.184 |
| ED length of stay (hours) | 0.00 | -0.02 to 0.01 | 0.604 |
| Site of enrollment |  |  |  |
| CUHC | Reference |  |  |
| BIDMC | 0.09 | -0.84 to 1.00 | 0.855 |

* Every one-point increase in the 5-item compassion measure composite score.

BIDMC, Beth Israel Deaconess Medical Center; CUHC, Cooper University Health Care; ED, emergency department.

**Supplemental Table 10**: Multivariable linear regression model with Healthcare System Distrust values subscale as the dependent variable (n = 794).

| **Variables** | **β Coefficients** | **95% CI** | **p-value** |
| --- | --- | --- | --- |
|  |  |  |  |
| Physician Compassion* | -0.33 | -0.44 to -0.22 | <0.001 |
| Nursing staff compassion* | -0.06 | -0.15 to 0.03 | 0.160 |
| Age (years) | -0.01 | -0.03 to 0.01 | 0.211 |
| ED length of stay (hours) | 0.00 | -0.01 to 0.00 | 0.335 |
| Site of enrollment |  |  |  |
| CUHC | Reference |  |  |
| BIDMC | -0.22 | -0.85 to 0.40 | 0.486 |

* Every one-point increase in the 5-item compassion measure composite score.

BIDMC, Beth Israel Deaconess Medical Center; CUHC, Cooper University Health Care; ED, emergency department.

**Supplemental Table 11**: Multivariable linear regression model with Healthcare System Distrust competence subscale as the dependent variable (n = 820).

| **Variables** | **β Coefficients** | **95% CI** | **p-value** |
| --- | --- | --- | --- |
|  |  |  |  |
| Physician Compassion* | -0.26 | -0.35 to -0.18 | <0.001 |
| Nursing staff compassion* | -0.19 | -0.26 to -0.12 | <0.001 |
| Age (years) | -0.01 | -0.02 to -0.00 | 0.021 |
| ED length of stay (hours) | 0.00 | -0.01 to 0.01 | 0.917 |
| Site of enrollment |  |  |  |
| CUHC | Reference |  |  |
| BIDMC | 0.19 | -0.22 to 0.61 | 0.362 |

* Every one-point increase in the 5-item compassion measure composite score.

BIDMC, Beth Israel Deaconess Medical Center; CUHC, Cooper University Health Care; ED, emergency department.

# III. Supplemental Figures

**Supplemental Figure 1**: Physician 5-item compassion measure items.


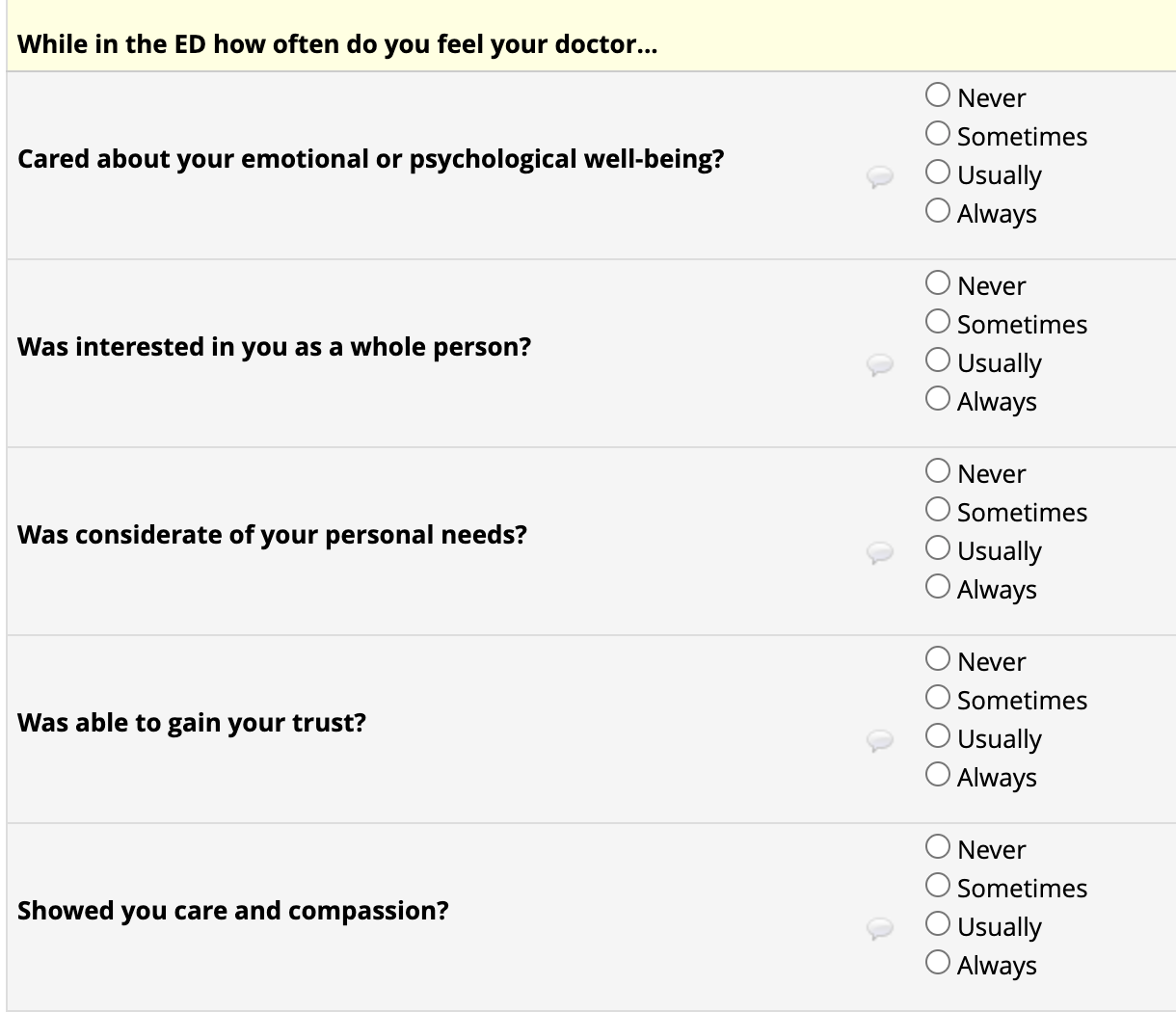


## **Supplemental Figure 2**: Nursing staff 5-item compassion measure items.


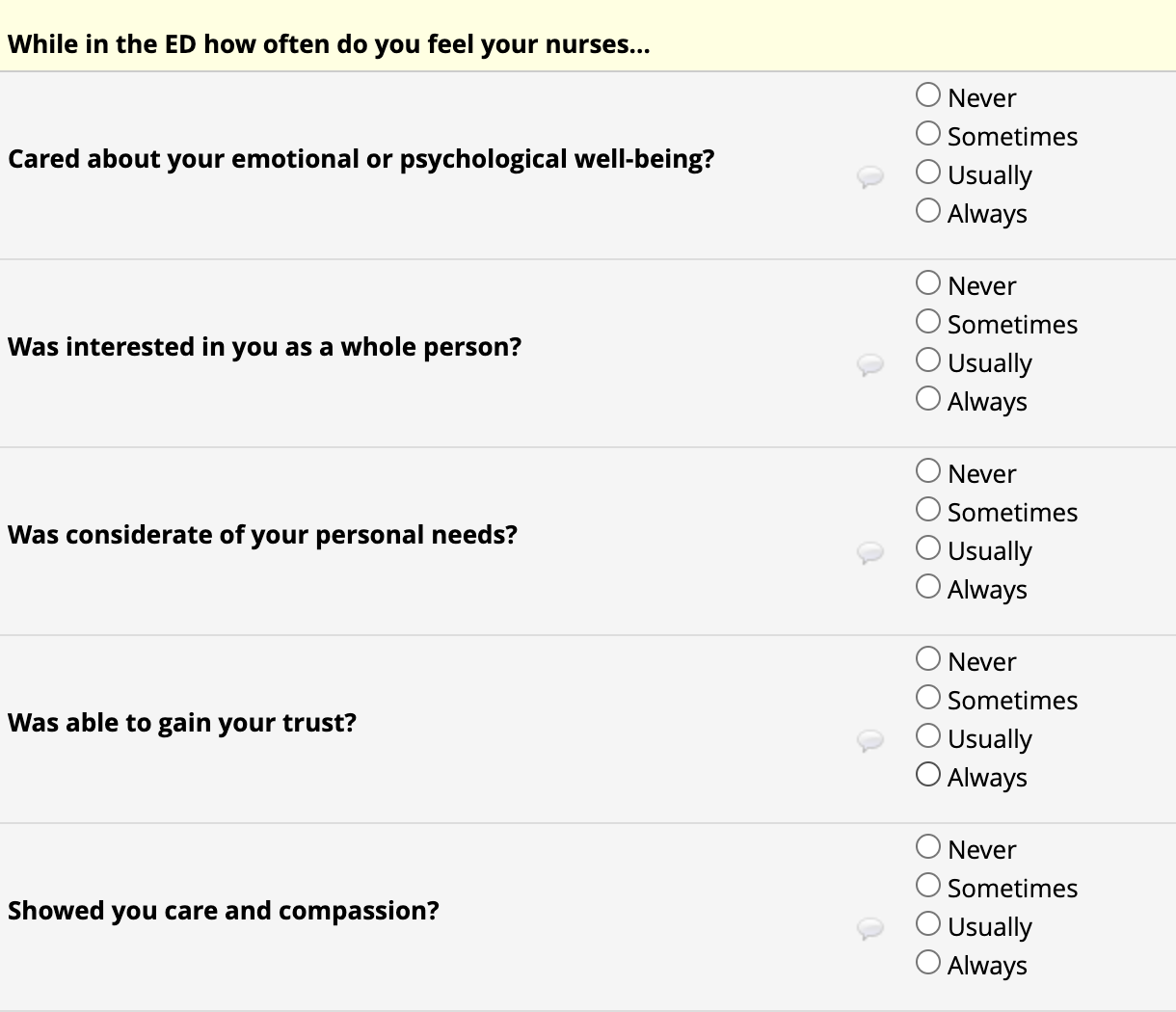


**Supplemental Figure 3**: Healthcare System Distrust Scale items.


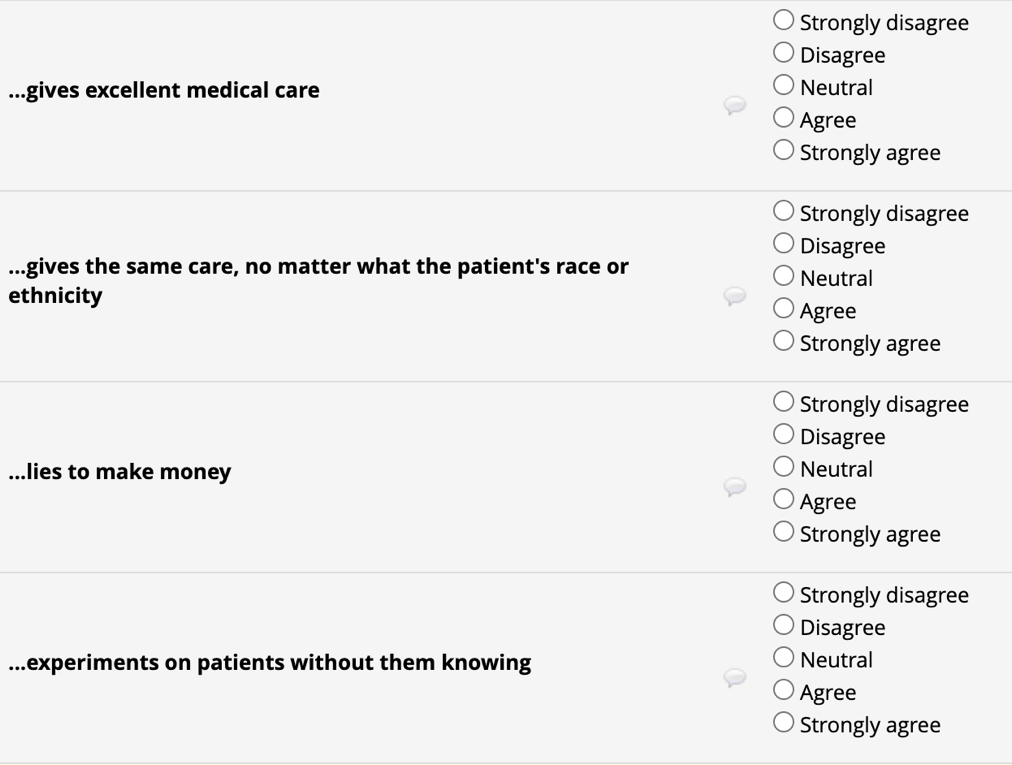


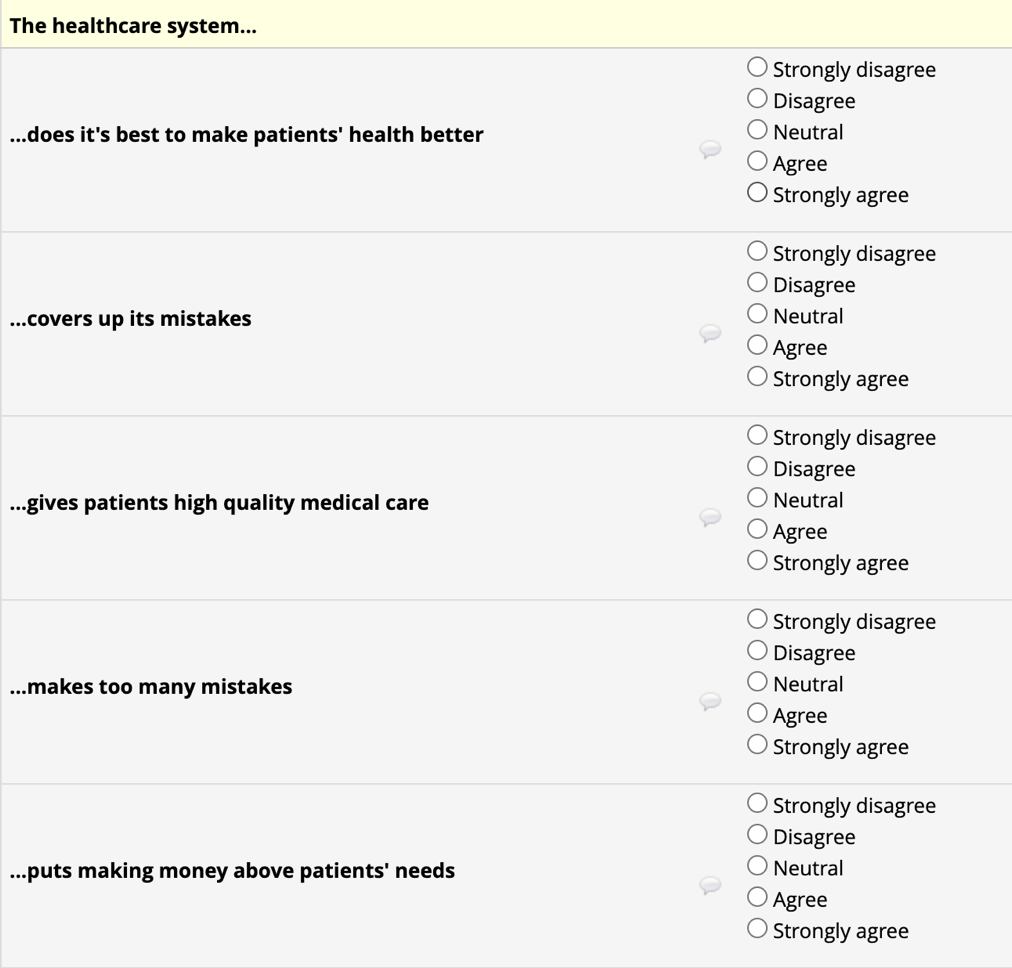


Items 1, 3, 6, and 7 are inversely coded. Values subscale included items 2, 5, 7, 8, and 9. Competence subscale included items 1, 3, 4, and 6

**Supplemental Figure 4**: Distribution of the physician 5-item compassion measure.

**Supplemental Figure 5**: Distribution of the nursing staff 5-item compassion measure.

**Supplemental Figure 6**: Distribution of the Healthcare System Distrust total scale.

**Supplemental Figure 7**: Distribution of the Healthcare System Distrust values subscale.

**Supplemental Figure 8**: Distribution of the Healthcare System Distrust competence subscale.
